# Wound of Abdomen, Expulsion of Intestines, and Cure

**Published:** 1846-12

**Authors:** J. Gibbs

**Affiliations:** Claiborne, La.


*Wound of Abdomen, Expulsion of Intestines, and Cure.* By J. Gibbs, M. D., Claiborne, La.—A labourer, 25 years of age, in a rencounter, on 9th Feb. 1846, received, with a dirk knife, a cut commencing opposite and two inches to the right of the navel, extending outwards and upwards, to the length of one inch and a half. The struggle did not cease until the jejunum, ileum and colon, together with the omentum, were expelled with serious lacerations to the latter. An incision of half an inch through the peritoneal coat of the small intestine was the only observable injury to the gut.

Two or three hours had elapsed before medical aid could be obtained, and now the patient lay prostrate and exhausted. The skin cold and moist, pulse feeble, nausea and ineffectual efforts to vomit, on the least motion. The gut was quite cold to the hand, and the large portion had assumed a dark purplish hue, and was badly swollen. Two small mangled portions of epiploon were removed with the scalpel; then by patient manipulation, the small gut was returned, securing it with the ulnar fingers of the left, pressing inwards with the thumb and index of the same, assisted by the right hand and thus staying it, whilst the first hold was being renewed.

The strangulation and tenderness of the colon required the incision to be enlarged some five or six lines before its reduction.

During the reduction, hemorrhage was profuse, and the patient suffered intensely, yet as no artery was discoverable, and the parts being brought in apposition, the interrupted suture, including seven or eight lines on either side, and the entire abdominal wall, save the peritoneum, with a pledget of lint, straps of plaster and bandage completed the dressing.

Quietude, horizontal position and farinaceous diet, being strictly enjoined, together with directions for giving an occasional anodyne and the mildest aperients, the patient was left quite disposed to sleep.

On the 11th the patient had lost blood occasionally for the first twenty-four hours, from the wound, on turning or exerting the abdominal muscles. Stools small and in slugs. Had slept. Skin and appetite improved. Pulse 74 and full.

Vs. to extent of ℥xxv.—(pulse rose to 100;) hydrarg. chlo. m. gr. iij, and repeated, constituted additional treatment.

On 25th, patient walking about, without having had scarcely an unfavourable symptom. The sutures, which now formed the only apparent injury, were removed and patient discharged.

In the above case, we have additional evidence of the passiveness of the stomach in vomiting, and the fallacy of dispensing with sutures in similar cases.

It should be remarked that the sutures used were large, and barely brought the lips in contact, allowing liberally for swelling and the exit of blood, and that, instead of aggravating, they appeared to perform the office of a salutary revulsive, obviating the wonted tumefaction in the lips of the wound, and the irritation, in the adjacent peritoneum, consequent on so much handling.—*Ibid.*

